# Correction to ‘MeCP2 binds to nucleosome free (linker DNA) regions and to H3K9/H3K27 methylated nucleosomes in the brain’

**DOI:** 10.1093/nar/gkac204

**Published:** 2022-03-22

*Nucleic Acids Research*, Volume 40, Issue 7, 1 April 2012, Pages 2884–2897, https://doi.org/10.1093/nar/gkr1066

Figures 1 and 2 show multiple signs of splicing. Different lanes of gels were brought together to make final figures. The splicing was done for cosmetic reason, without intent to deceive, and is visible to the naked eye.

New figures are provided below.

These corrections do not affect the interpretation of the results and conclusions of the article.


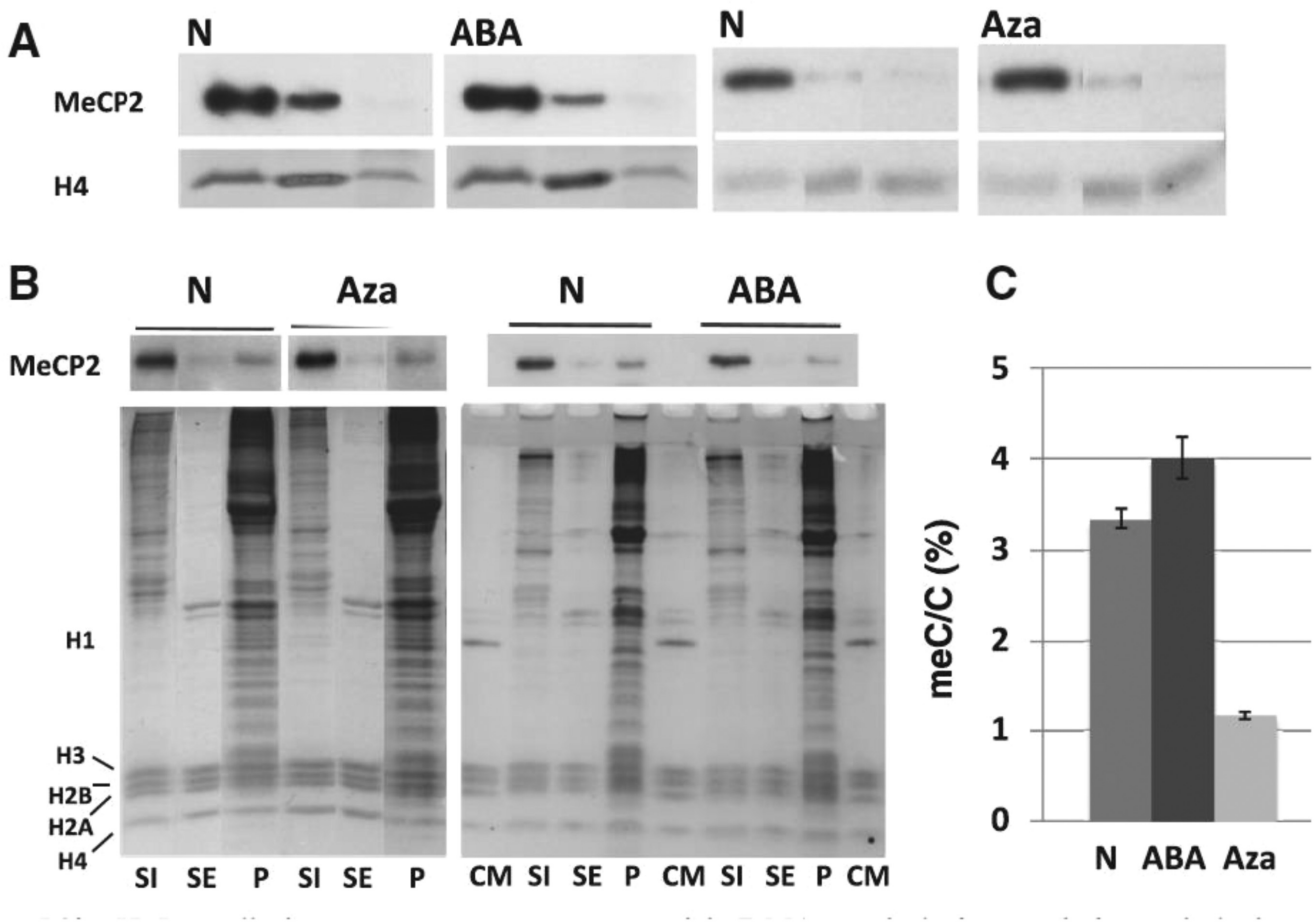


**New Figure 1**.


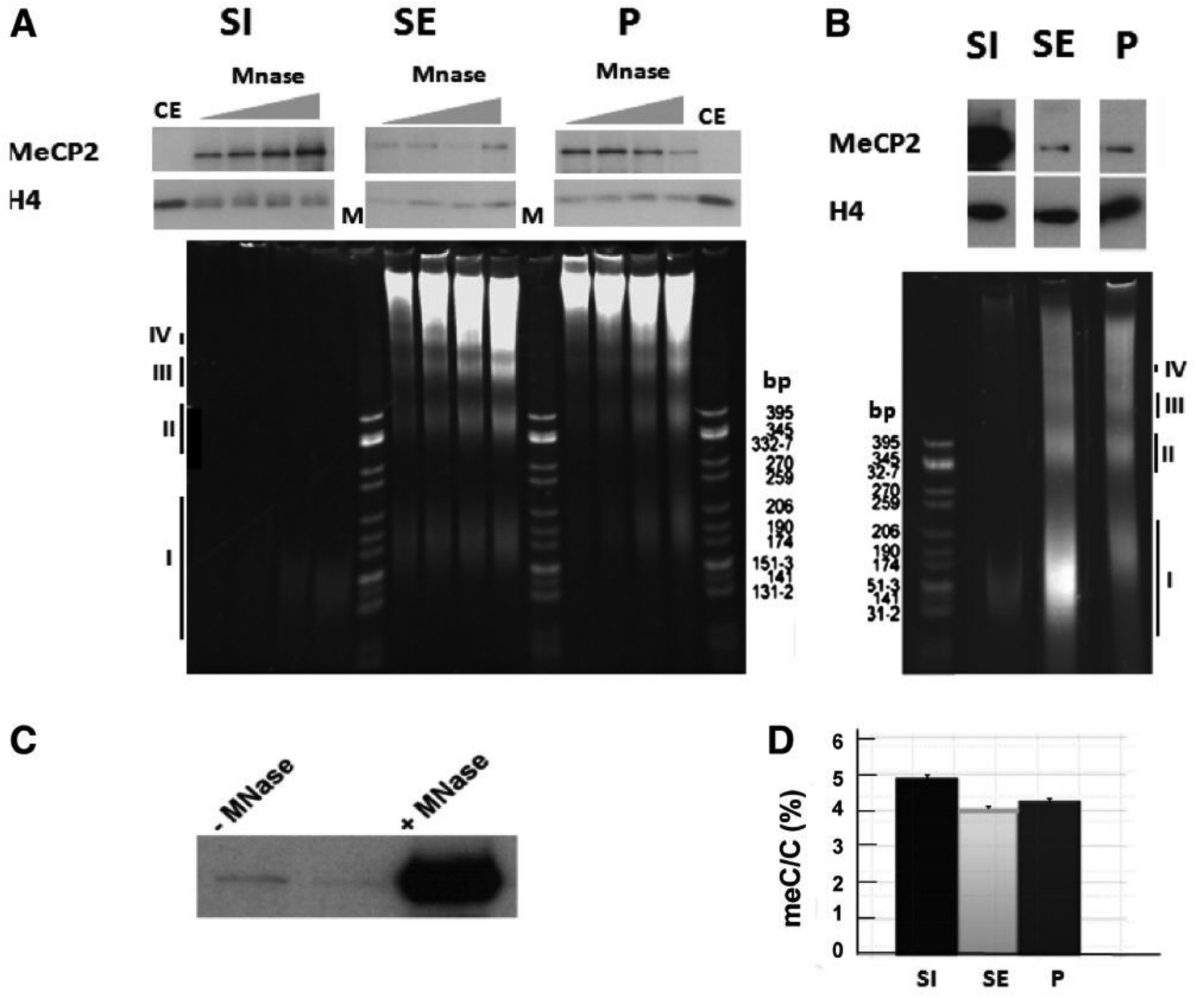


**New Figure 2**.

